# Nutritional Composition and Phytochemical, Antioxidative, and Antifungal Activities of* Pergularia tomentosa* L.

**DOI:** 10.1155/2017/6903817

**Published:** 2017-03-20

**Authors:** Imen Lahmar, Hafedh Belghith, Ferjani Ben Abdallah, Karima Belghith

**Affiliations:** ^1^Research Unit in Plant Biodiversity and Ecosystem Dynamics in Arid Environment, Sfax Faculty of Sciences, Sfax, Tunisia; ^2^Laboratory of Molecular Biotechnology of Eukaryotes, Center of Biotechnology of Sfax, Sfax, Tunisia; ^3^Laboratory of Plant Biotechnology Applied to Crop Improvement, Sfax Faculty of Sciences, Sfax, Tunisia

## Abstract

Crude extracts from a medicinal Tunisian plant,* Pergularia tomentosa* L., were the investigated natural material. Butanolic extract of roots analyzed with IR spectra revealed the presence of hydroxyl, alcoholic, and carboxylic groups and sugars units. Analysis of some secondary metabolites, total phenolic, flavonoids, flavonols, and procyanidins, was performed using different solvents following the increased gradient of polarity. Fruits and leaves contained the highest amounts of all these compounds. Antioxidant properties were evaluated by the determination of free radical scavenging activity and the reducing power of methanolic extracts. Fruits and leaf extracts were the most powerful antioxidants for the two-assay in vitro system. Stems and fruits extracts exhibit an antifungal activity against* Fusarium oxysporum f. sp. lycopersici* which could become an alternative to synthetic fungicide to control* Solanum* species fungal diseases.

## 1. Introduction

Bioactive molecules obtained from medicinal wild plants and their curative potentials are well documented [1]. Recent studies showed that a large number of herbs products including polyphenolic substances can be considered as the most abundant plant secondary metabolites with highly diversified structures. This source of phytochemicals was related to a variety of biological activities including antioxidant potential [2]. Oxidative stress and cell damage occur in the human body when the Reactive Oxygen Species were in imbalance with the antioxidants. Those compounds were associated with cardiovascular disease, neurological disorders cancer, diabetes, and other diseases [3]. The use of synthetic antioxidants presents undesirable effects on health on long-term life [4]. Due to the fact that they have been considered as a major group of chemicals contributing to the antioxidant potential of plant extracts, phenolic compounds present in medicinal plants could be consumed in the diet and they have limited or no side effects [5, 6]. Their ingestion reduces the risk of certain cancers and reverses the human oxidative damage by acting as exogenous antioxidant system [7, 8].

Plants rich in phenols, flavonoids, tannins, vitamins, and many more phytochemicals were searched for the development of ethnomedicines and were responsible for several pharmacological activities [9]. Phytochemicals extracted from plants have been recognized as some of the most promising compounds for the development of novel ecofriendly compounds due to their high degradability and low incidences of negative impacts in comparison with synthetic fungicides chemicals giving rise to residual toxicity, hormonal imbalance, and carcinogenicity [4, 10]. As phytopathogenic fungi,* Fusarium* spp. is the largest expansion in “Tuberculariaceae” family. It causes wilts and cankers, stalk rots, leaf wilting, maize horticultural, field, and ornamental plants and affects tomato and many cucurbits [11, 12].

*Pergularia tomentosa* L., commonly known as Bou Hliba in Tunisia, is a fetid smelling lactiferous twinner [13]. At the slightest touch, leaves and fruits secret a sticky white fluid [14]. The plant was widely distributed across the Horn of Africa [15] to Sinai, Jordan, and Saudi Arabica [16]. Crushed* P. tomentosa* was administered in the case of diarrhea and the sap of leaves was used as ocular instillation and regarded as a sovereign remedy for the ills of the head [17]. The roots were used for the treatment of bronchitis, constipation, and skin diseases and leaves for bronchitis and tuberculosis [18]. In Egypt, the plant was used as a poultice, depilatory, laxative, antihelmintic, and abortifacient [19]. The latex of stems and leaves irritates the skin and eyes and can cause inflammation [20]. The plant was reported to contain cardiotonic glycosides such as desglucouzarin, coroglaucigenin, and uzarigenin in the leaves [21]. Roots contains uzarigenin, pergularoside, ghalakinoside, and calcatin and their derivates, 6′-hydroxycalactin, 6′-hydroxy-16*α*-acetoxycalactin, 16*α*-hydroxycalactin [22], 3′-O-*β*-D-glucopyranosylcalactin, 12-dehydroxyghalakinoside, and 6′-dehydroghalakinoside [23]. The plant was reported to have molluscicidal activity [24] and its cardenolides induce cell death by apoptosis of Kaposi in the case of cancer [23].

The screening of the nutritional composition as the relevance of the presence of phytochemical and antioxidative potentials in a wild plant can lead to valorizing its implication in human diet, animal fodder, and sol fertilization. The fact that it can have antifungal activity affects beneficially the health of the product consumer and the economic capacity of the farmer. In this context, the present studies aimed to provide the nutritional composition and phytochemical analysis of principal polyphenols extracted from* Pergularia tomentosa* L., a therapeutically important medicinal plant. The antioxidant properties of four different organs (roots, stems, leaves, and fruits) were explored using a set of in vitro antioxidant assays including scavenging of DPPH as well as reducing power assay. Antifungal activity against* Fusarium oxysporum f. sp. lycopersici* was tested for the extracts obtained by the fractional method following an increased gradient of polarity.

## 2. Materials and Methods

### 2.1. Plant Materials

*Pergularia tomentosa* was collected from the surroundings of the region of Bïr Ben Ayed (south Sfax, Tunisia, arid climate). It was growing in the borders of olives farms. The collection was in early morning in the beginning of March. Plants were in flowering stadium and have simultaneously green and mature fruits. Handling of the sample amount from the farm was manually and was done by wearing plastic gloves to protect the skin from the latex secreted by fruits, stems, and leaves at the slight touch. The specimen was identified at the Herbarium of the Sfax Faculty of Sciences by Professor Ben Abdallah Ferjani. Roots, stems, leaves, and fruits were finely ground using an electric blender and stored in plastic containers at room temperature and in darkness until required for use.

### 2.2. Reagents

2,2-Diphenyl-1-picrylhydrazyl (DPPH), butylated hydroxytoluene (BHT), *α*-tocopherol, Folin-Ciocalteu reagent, potassium ferricyanide, and sodium carbonate were purchased from Sigma-Aldrich, USA. All solutions were freshly prepared in distilled water. The solvents used for extraction and partition were from Sigma-Aldrich.

### 2.3. Morphological and Phenological Characterizations

Morphological characterization was determined by simple observation. Physical characteristics of the whole plant, even for each organ, were determined on 10 samples taken at random. The dimensions of the different organs were determined using a caliper. Weights of fruits and seeds were determined using an analytical balance of an accuracy of ±0.001.

### 2.4. Preparation of Different Extracts

#### 2.4.1. Infusion

To estimate protein content, pH, titratable acidity, total sugars, reducing sugars, phenolic compounds, and antifungal activity, aqueous extracts were prepared. 5 g of the dry matter was incubated in bowling water for 15 to 20 min. The filtrate was retained and the infusion was repeated three times. The totality of filtrates was lyophilized.

#### 2.4.2. Extraction following Increased Polarity

**Figure 1:**
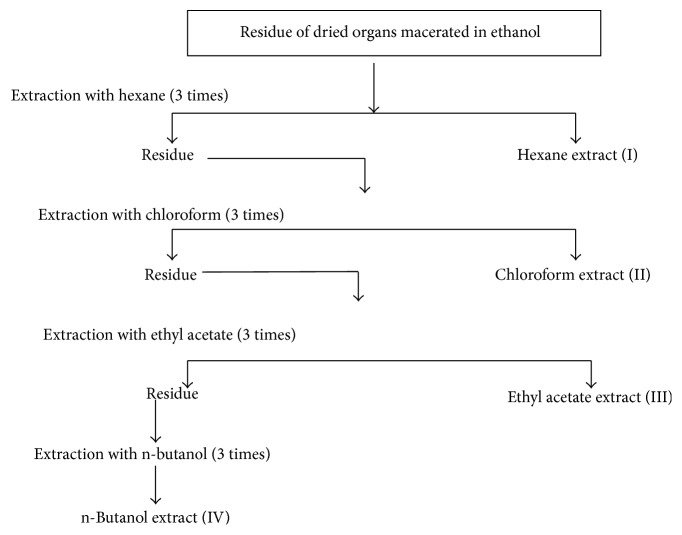
Diagram of fractional extraction of phenolic compounds from roots, stems, leaves, and fruits of* Pergularia tomentosa* L.

To extract phenolic compounds, each organ was macerated with ethanol. 50 g of was macerated with 200 mL of 80% ethanol during 48 hours at 40°C and with continuous agitation. The residue obtained after the filtration of the mentioned mixture was extracted twice with the same solvent and at the same conditions. This mixture was filtrated and the obtained filtrate was evaporated using a rotary evaporator at 40°C until discarding the totality of ethanol. The obtained residue after evaporation under reduced pressure was partitioned following an increased gradient of polarity, with successively hexane, chloroform, ethyl acetate, and n-butanol (Figure 1). Only the n-butanol extract from stems showed two phases: organic and aqueous.

#### 2.4.3. Extraction with Methanol

To determine the antioxidant activity, the extraction was performed with methanol. 50 g from each ground plant organ was soaked in pure methanol. Extraction was repeated three times, considering every time that the plant material should be submerged wholly by the sufficient quantity of fresh methanol. Each extraction lasts 3 days with continuous agitation in shaker at 37°C. All the combined extracts were concentrated on a rotary evaporator under vacuum at 40°C. To evaluate the antifungal activity of* P. tomentosa,* extracts I, II, III, and IV of stems, leaves, roots, and fruits (Figure 1) were used in addition to the aqueous extract of each organ.

To compare the proportions sample/extract, the extraction yield was calculated according to the following formula:

(1) Yield%=residu weight obtained after solvent evaporationinitial sample weight×100.

### 2.5. Screening of Nutritional Composition

The moisture content was determined by drying in an oven at 103 ± 2°C (NF V05-108, 1970). The protein content was estimated by the Kjeldhal method [25] based on the following equation:

(2) $$\begin{array}{c} N\left(\;\%\;\right)=\frac{\left(N-N^{\prime }\right)\times 0.05\times 1.4\times \left(V/V^{\prime }\right)}{P}, \end{array}$$

where *V* is volume of the mineralized solution (mL), *V*′ is added volume of sodium solution (mL), *N* is read amount of sulfuric acid after titration (mL) with the sulfuric acid (0.05 N), *N*′ is volume of sulfuric acid added after control titration (mL), and *P* is sample weight (g).

The amount of proteins was calculated by the multiplication of the rate of the total azote *N* (%) by the coefficient 6.25.

Total fat was evaluated by the Soxhlet method (NF EN ISO 734-1, 2000) using hexane as a solvent for the extraction. The ash content was determined by the incineration of samples in a muffle furnace at 550 ± 5°C until a whitish color (NF V 05-113, 1972). Total carbohydrates were calculated as the residual difference after subtracting proteins, ash, moisture, and lipid content [26]. Total energy (nutritive value) of each sample was estimated in kcal by multiplying the values obtained for protein, fat, and available carbohydrate by 4.00, 9.00, and 4.00, respectively, and by adding up the values [27]. The pH was measured according to the potentiometric method (NT 52.21, 1982). The titratable acidity was determined in the presence of sodium hydroxide (0.1 N) for the titration and phenolphthalein as a color indicator (NF V 05-101, 1974). The titratable acidity was expressed in acetic citric g/100 g sample and based on the following equation:

(3) $$\begin{array}{c} A\left(\;\%\;\right)=\frac{250\times V_{1}\times 0.07}{V_{0}\times W\times 10}\times 100, \end{array}$$

where *W* is sample weight (g), *V*_0_ is volume of the test sample (mL), *V*_1_ is volume of the hydroxide sodium solution at 0.1 N (mL), and* 0.07* is conversion coefficient of the titratable acidity as equivalents of citric acid.

Total sugars were assayed by the phenol-sulfuric method based on the absorbance at 490 nm of phenol-carbohydrate complex [28]. The calculation of their amount was based on the equation of the calibration curve: *y* = 0.434*x* (determination coefficient *R*^2^ = 0.972). Reducing sugars were estimated with 3,5-dinitrosalicylic acid (DNS) [29]. The amount of reducing sugars was expressed in g/L, with a calibration curve equation *y* = 0.921*x* (determination coefficient *R*^2^ = 0.986).

### 2.6. Infrared Spectroscopy

Aqueous phase of the n-butanol extract of stems was pressed into pellets for the estimation of the infrared spectra with the scanned wave ranging from 4000 to 500 cm^−1^. Spectra were recorded on a Perkin Elmer Universal ATR Sampling Accessory.

### 2.7. Polyphenol Content

Folin-Ciocalteu method based on the reduction of a phosphotungsten-phosphomolybdate complex was used to determine phenolic compounds [30]. 50 *μ*L of Folin-Ciocalteu reagent (2 N) was added to 800 *μ*L of extract. After 3 min of incubation at room temperature, 150 *μ*L of sodium carbonate solution was added. The absorbance was measured at 765 nm after 2 h. Total phenolic content was expressed as gallic acid equivalents (GAE) in mg/g dry weight and using the following equation based on the calibration curve: *y* = 0.0864*x*, where *y* is the absorbance and *x* is the concentration (determination coefficient: *R*^2^ = 0.985).

Total flavonoids content of the extracts was determined spectrophotometrically [31], using a method based on the formation of the complex flavonoid-aluminum having a maximum absorption at 430 nm. After addition of 2 mL of 2% aluminum chloride to each extract (1 mL), the mixture was incubated for 15 min. Flavonoid content was expressed as quercetin equivalents (QE) in mg/g dry weight and following the equation based on the calibration curve: *y* = 0.0301*x* (determination coefficient: *R*^2^ = 0.983).

Flavonols were estimated by the aluminum chloride method [32]. Aliquots were prepared by mixing of 1 mL of extracts and 1 mL of aqueous aluminum chloride (20% in ethanol). The absorbance was determined at 425 nm against a blank. The results were expressed as quercetin equivalents (QE) in mg/g dry weight and using established equation from the calibration curve: *y* = 0.035*x* + 0.001 (determination coefficient: *R*^2^ = 0.992).

The amount of condensed tannins (procyanidins) was measured using the modified vanillin assay for an absorbance measured at 500 nm [33]. The results were expressed as catechin equivalents (CTE) in mg/g dry weight and using the following equation based on the calibration curve: *y* = 0.238*x* (determination coefficient: *R*^2^ = 0.998).

### 2.8. Antioxidant Activity

#### 2.8.1. Evaluation of DPPH Scavenging Activities

The radical scavenging assay of methanolic extracts was measured as equivalent of hydrogen-donating, according to DPPH method [34] with some modifications. Briefly, 1 mL of DPPH solution (10^−4 ^M) was added to 1 mL of sample solution at various concentrations (0.0625–2 mg/mL) and the pH was adjusted to 7.4. The change in color from deep violet to light yellow after 20 min of incubation in dark was measured at 517 nm against a control. The inhibition percent was calculated according the equation below:

(4) Radical scavenging effect%=1−optical absorbance of the sampleoptical absorbance of the control×100.

Extract concentration providing 50% inhibition (IC_50_) was calculated from the plot of scavenging effect (%) against the extract concentration. In the linear regression of plots, the abscissa represented the concentration of the tested plant extracts and the ordinate represented the average percent of scavenging capacity from three replicates. BHT and *α*-tocopherol were used as positive control.

#### 2.8.2. Reducing Power Assay

The reducing power of the methanolic extract of* P. tomentosa* was determined according to the method previously described by [35]. This method was based on the colorimetric change of color when Fe^3+^ transforms to Fe^2+^ at 700 nm. 1 mL of extract solution at different amounts was mixed with 2.5 mL of 0.2 M phosphate buffer (pH 6.6) and 2.5 mL of 1% potassium ferricyanide. The obtained reaction mixture was then incubated in a water bath at 50°C for 20 min. 2.5 mL of 10% of trichloroacetic acid was added to the mixture. The upper layer solution (2.5 mL) obtained after centrifugation at 3000 was mixed to 2.5 mL of deionized water and 0.5 mL of fresh ferric chloride (0.1%). Increased absorbance of the reaction mixture indicates increased reducing power. The sample concentration providing 0.5 of absorbance (IC_50_) was calculated by plotting absorbance against the corresponding sample concentration. BHT was used as a standard.

### 2.9. Antifungal Activity

*Fusarium oxysporum f. sp. lycopersici* was grown on plates of Potato Dextrose Agar (PDA) at 30°C for approximately 1 week to be well sporulated before testing. The inoculum was prepared by scraping the superficial mycelium and directly suspending the fungal material in water. The resulting suspension was then filtered through sterile gauze to obtain a homogeneous suspension of small hyphae. The CFU (Colony Forming Unit) of the inoculums suspension of* Fusarium* was approximately 0.4 × 104 to 5 × 104 CFU/mL. Tests were carried out by the microdilution method [36]. The microdilution test was performed by using sterile and multiwell microplates (96 U-shaped wells). The two times extracts concentrations were dispensed into the wells of rows 1 to 10 of the microdilution plates in 100 *μ*L volumes diluted with RPMI 1640 medium. After 24, 48, and 72 hours of incubation, the MIC_0_, MIC_1_, MIC_2_, and MIC_3_ were noticed for each concentration of the designed extract. The MIC (Minimal Inhibitory Concentration) was defined as the lowest extract concentration that substantially inhibits visually growth as detected when testing the antifungal agent. In addition to the extracts obtained by different solvents, we prepared aqueous extracts for each organ of the plant.

### 2.10. Statistical Analyses

Independent samples of each item were analyzed in triplicate and data were presented as mean ± SD (standard deviations) and the confidence limits were set at *p* < 0.05 for all values.

## 3. Results and Discussion

### 3.1. Morphological Characterizations

Opposite leaves having dimensions of 14.25 to 46 mm of length and 20 to 52.7 mm of width present petioles of 13 to 25 mm length. Branching stems are cylindrical, climbing, and pubescent with small gray hairs along their length. Roots are pivotal and fairly rigid. They are deep and have bristly and short root-hair. The fruits are elongated follicles of 24.5 × 60 mm. They are in two halves follicles assembled together, rarely found in the form of three or four, and each of them contains 31 to 50 seeds. Seeds are ovoid, flattened, and compressed against each other. They are 4 × 9.2 mm and weigh 0.8 to 1.1 mg. At maturity, they are surmounted at their point by a tuft of white hairs of 3 to 4 cm being detached by the wind. The fragrant flowers grouped into false-umbels. The calyx is glabrous and purple, while the corolla is whitish-yellow with five linear divisions which are acute, reflected, and rolled at their margins.

### 3.2. Proximate Analysis and Nutrients Composition

The study of the nutritional content (Table 1), for instance, the moisture content presented in the different parts of the plant, could reflect its ability to resist the environmental conditions and to adapt to drought as, by the continuous personal observations of* Pergularia tomentosa* in its ecological habitat, it was found that it remains practically all the year green and maintains the health aspect. The percentages in ash content in leaves and fruits could be involved in important nutritional mineral elements [37]. The content of carbohydrate in roots was higher in comparison with the peel of* Picralima nitida* (37.7 ± 0.18%) [38] and so lower than the seeds of* Saba florida* (79.56 ± 0.033%) [39]. Calculated total energy in that* P. tomentosa* may encourage its use as a source of feed and fodder for animals and/or of possible human use in addition to its medicinal properties. Titratable acidity, especially low in leaves and roots, can be responsible for the proliferation inhibition of the microbial flora in the plant organism. The increased pH and the decrease in titratable acidity that occurs with a degree of maturity can be affected by the loss of citric acid. The wealth of total and reducing sugars in stems and roots allows the maintaining of the turgor and the cytosolic volume and the preservation of the membrane integrity of dried organs [40]. The different variations registered may depend on ecological factors, geographic distribution, plant age, cultivation climatic conditions, and vegetative cycle as well as the adopted mechanism of adaptation to the new condition of* P. tomentosa*.

### 3.3. Extraction Yield

The obtained yield of each sample relative to the dry weight as well the methods of extraction was shown in Table 2. The results showed that there is a significant variation in the extraction yield between solvents for each plant organ. Concerning the increasing gradient of polarity, the n-butanol extract seems to have the more important extraction yield followed by the hexane extract of leaves and fruits and the chloroform extracts of roots and stems. However, extraction yields of aqueous and methanolic samples were higher in comparison with those of fractional extraction. It seems that the extraction yield depends on the solubility degree of the compounds in the used solvent during the extraction manipulation. The extraction at room temperature and with continuous agitation may lead to extract the maximum of bioactives compounds and prevent their degradation. A certain temperature degree can inactivate bioactives compounds and decrease their extraction yield in the used solvent.

### 3.4. Infrared Spectra

Only the n-butanol extract (IV) of stems represents two phases: aqueous and organic. The first phase was analyzed by IR.

**Figure 2:**
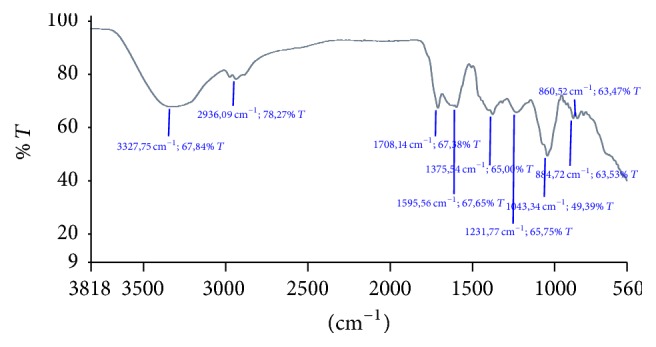
Infrared (IR) spectra of the aqueous phase of the n-butanolic extract of stems of* Pergularia tomentosa*.

In IR spectra (Figure 2), the bands at 3327.45 cm^−1^ and 2936.09 cm^−1^ correspond to the hydroxyl group of alcohols. The peak around 1708.14 cm^−1^ was due to stretching vibrations of C=O of esters and ketones which were abundant practically in the same region (1680–1740 cm^−1^) [41]. While the band at 1598.56 cm^−1^, of low intensity, indicates the vibrations of the C=C aromatic compounds, the band at 1375.54 cm^−1^ confirmed the presence of the aliphatic chain CH_3_. The more intense absorptions at 1043.34 and 1231.77 cm^−1^ could be attributed to stretching vibrations of C-OH side groups and the C-O-C glycosidic band vibration. Peaks located at 884.72 and 860.52 cm^−1^ indicate, respectively, *β*-configuration and *α*-configuration of the sugar units [42].

### 3.5. Analysis of Phenolic Compounds

**Figure 3:**
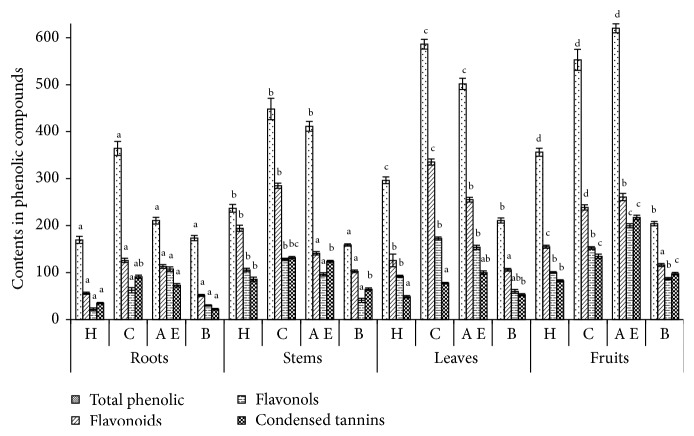
Amounts of total phenolic (mg Eq GAE/g DW), flavonoids (mg Eq QE/g DW), flavonols (mg Eq QE/g DW), and condensed tannins (mg Eq CTE/100 g DW) of different extracts of* Pergularia tomentosa*. H: hexane; C: chloroform; A E: ethyl acetate; B: n-butanol; mg Eq GAE/g DW: mg of gallic acid equivalents per g of dry weight; mg Eq QE/g DW: mg of quercetin equivalent (QE) per g of dry weight; mg Eq CTE/100 g DW: mg catechin equivalents (CTE) per g dry weight. The values are the mean of three determinations ± standard error. The different lowercase letters represent significant differences between samples versus different solvents by Student's *t*-test (*p* < 0.05). Values for the same compound and the same solvent, not sharing a common letter (a, b, c, or d), differ significantly at *p* < 0.05.

The phenolic compounds were commonly involved in the prevention of many cancers and in the defense against microbial invasion [4]. The total phenolic, flavonoid, flavonol, procyanidins, and gallotannin content (Figure 3) show differences in amount depending on solvents polarities. The highest amount was found in ethyl acetate extracts of fruits and in chloroform extracts of leaves followed by chloroform-fruits and ethyl acetate-leaves.

For the four organs, the important values of different phenolic compounds were unregistered for both chloroform and ethyl acetate. Moreover, high solubility of phenols in polar solvents provides a high concentration of these compounds in the extracts [43]. These amounts were higher than the concentration registered in the leaves of* Convolvulus arvensis* (244.6 ± 2.9 mg GAE/g DW) [44]. The synthesis of polyphenols may likely be influenced by environmental conditions (temperature, sunlight exposure, dryness, and salinity), genetic difference, also to the time of collection, and the suitable method of storage.

Flavonoids have antiviral, antitumoral, and anti-inflammatory properties [4]. Extracts of chloroform-leaves and chloroform-stems contain the high amounts of flavonoids (Figure 3). The important content of flavonol was found in acetate ethyl-fruits extract and chloroform-leaves extract whereas hexane and n-butanol extracts have the lowest amounts. These results were in accordance with the literature and they depend on the polarity of solvents used in the extraction [45]. Procyanidins present a defense against attacks from predators such as insects and herbivorous [46]. Fruits and stems showed the highest levels of condensed tannins.

### 3.6. Antioxidant Activity

**Figure 4:**
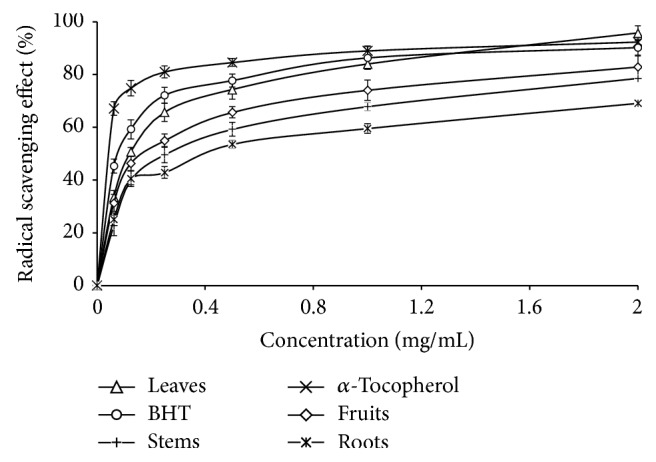
Radical scavenging effect (%) on DPPH (2,2-diphenyl-1-picrylhydrazyl ) radicals of* Pergularia tomentosa*'s organs. The values are the mean of three determinations ± standard error.

Antioxidant activity can depend on many factors such as the lipid composition, the antioxidant concentration, and the kind of plant. The antioxidant capacity of* Pergularia tomentosa* samples was reported to be highly dependent on the composition of these extracts and on the manipulation conditions during in vitro tests. Following DPPH method, the activity was evaluated by determining the IC_50_ value, corresponding to the concentration of the extract that was able to inhibit 50% of the free radicals. Under the assay conditions (Figure 4), among the four extracts, the most potent antioxidant activity was detected in leaves extract. The powerful antioxidant activity of leaves and fruits extract can be attributed mainly to the phenolic content, due to their hydroxyl groups, and/or to flavonoids which react with the DPPH radical by hydrogen atom donation to free radicals [47], while a highly positive correlation between total phenolic content and antioxidant activity was established in case of many plant species [48, 49].

**Figure 5:**
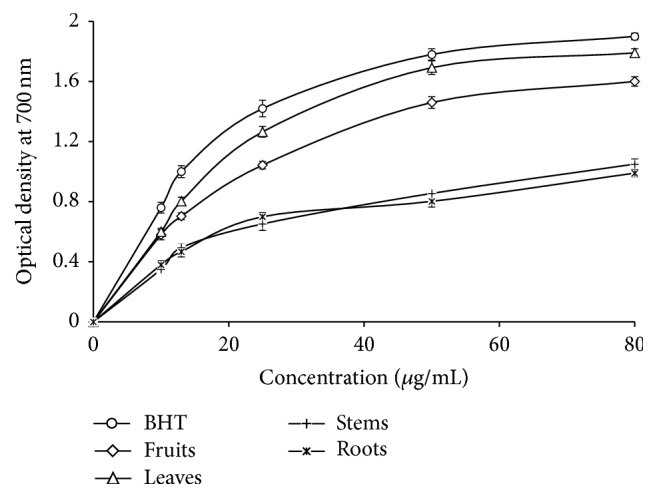
Reducing power of methanolic extracts of* Pergularia tomentosa*, as measured by changes in optical density at 700 nm. The values are the mean of three determinations ± standard error.

The assessment of antioxidant activity by reducing iron represents the ability of a substance to transfer an electron or a hydrogen atom from another substance and an antioxidant ability to reduce the oxidized intermediates during the peroxidation processes [50].  Figure 5 shows the plot of reducing power of* P. tomentosa* methanolic extracts with the concentration range of 10–80 *μ*g/mL and comparing with BHT as a reference antioxidant. Ferric reducing antioxidant power was proportional to the increase of the concentrations of extracts. Leaves and fruits seem very potent. Roots representing the highest IC_50_ imply the lowest reducing power. The reducing activity of fruits and leaves was attributed to the presence of phenolic compounds that may act by donating the electrons and reacting with free radicals to convert them to more stable products and terminate radical chain reaction [51]. A positive correlation was established between antioxidant activities and reducing power with a determination coefficient *R*^2^ = 0.8812. However, the reducing power of an extract can be used as a significant indicator of its antioxidant activity [52]. Another positive correlation was established. The extracts with the highest levels of phenolic compounds also showed the highest antioxidant activity which is consistent with the literature [53].

### 3.7. Antifungal Activity

*Fusarium oxysporum f. sp. lycopersici* was responsible for the most important and widespread disease of the tomato fields by causing root-rot and vascular diseases on the plants [54]. After 72 h, the observation of the wells reveals that only aqueous extracts of stems and leaves, n-butanol extract of fruits, and ethyl acetate extract of fruits showed positive results (Table 3). In the case of ethyl acetate extract of fruits, the total inhibition action is higher than 2 mg/mL and 75% of* Fusarium* can grow in the presence of 1 mg/mL of* P. tomentosa* extract. Fruits extracted with n-butanol constitute the most potent effective fungicide with a minimum concentration of 0.25 mg/mL. Aqueous extract of stems has a total inhibitory value of the fungi growth at a concentration higher than or equal to 20 mg/mL. Aqueous extract of leaves shows an inhibition of 25% of the fungal growth at 20 mg/mL. Those four extracts could be used as a cheaper natural source to have minimal environmental impact and danger to consumers of tomato in contrast to synthetic pesticides. Our results were considered promoted compared with* Acorus calamus* which showed the highest antifungal activity at 1000 mg/mL [55]. The differences in the inhibitory effect of various plant extracts may be due to qualitative and quantitative differences in the present antifungal compounds and to the used solvent for extraction. Fruits and stems seemed to contain important metabolites resistant to the fungal growth.

The mechanism of action against pathogens can be explained by the fact of production of an enzymatic inhibition by phenols through compound oxidation and synthesis protein inhibition in the cell by tannins [56]. A synergistic interaction between extract and antimicrobial agents was recorded by Adwan et al. [57]. A survey against our studied* Fusarium* attributed the antifungal activity to the presence of some compounds, such as ethyl iso-allocholate; 7,8-epoxylanostan-11-ol; and 3-acetoxy [58].

## Figures and Tables

**Table 1:** Nutritional values of different organs of *Pergularia tomentosa*. DW: dry weight of plant. Cal: calorie. The values are the mean of three determinations ± standard error.

Parameters	Roots	Stems	Leaves	Fruits
Moisture (%)	11.12 ± 0.56	13.02 ± 0.47	15.31 ± 0.79	23.1 ± 0.85
Ash (%)	6.72 ± 0.75	8.55 ± 0.42	19.44 ± 0.51	15.73 ± 0.68
Protein (%)	3.67 ± 0.13	5.14 ± 0.2	5.72 ± 0.26	6.89 ± 0.33
Lipids (%)	2.51 ± 0.25	3.53 ± 0.18	4.63 ± 0.19	5.46 ± 0.26
Carbohydrates (%)	76.58 ± 0.52	70.62 ± 0.38	55.82 ± 0.71	49.93 ± 0.49
Total energy (kcal/100 g)	343.59 ± 0.3	334.81 ± 0.25	287.83 ± 0.39	276.42 ± 0.36
pH	7.23 ± 0.13	6.41 ± 0.09	7.86 ± 0.07	6.47 ± 0.23
Titratable acidity (% citric acid)	0.031 ± 0.004	0.127 ± 0.02	0.029 ± 0.006	0.08 ± 0.007
Total sugars (g DW/L)	20.35 ± 0.15	22.12 ± 0.09	15.37 ± 0.11	19.98 ± 0.12
Reducing sugars (g DW/L)	1.56 ± 0.13	0.74 ± 0.12	0.27 ± 0.07	1.55 ± 0.09

**Table 2:** Extract yield (%) from *Pergularia tomentosa* organs with different solvents. The values are the mean of three determinations ± standard error.

	Hexane	Chloroform	Ethyl acetate	n-Butanol	Water	Methanol
Roots	1.96 ± 0.03	3.4 ± 0.07	0.48 ± 0.2	3.64 ± 0.05	14.56 ± 0.51	17.23 ± 0.33
Stems	1.62 ± 0.01	0.72 ± 0.1	0.68 ± 0.06	6.51 ± 0.25	18.2 ± 0.29	25.11 ± 0.7
Leaves	4.34 ± 0.12	3.71 ± 0.02	0.98 ± 0.09	9.16 ± 0.34	22.71 ± 0.62	28.6 ± 0.58
Fruits	4.32 ± 0.08	1.02 ± 0.18	1.7 ± 0.13	8.66 ± 0.98	13.2 ± 0.34	27.55 ± 0.84

**Table 3:** Minimal Inhibitory Concentration (MIC) of *Pergularia tomentosa* extracts against *Fusarium oxysporum f. sp. lycopersici*.

Extract	Concentration (mg/mL)							
	2	1	0.5	0.25	0.125	0.063	0.032	0.016
n-Butanol, fruits				MIC_0_		MIC_1_	MIC_3_	
Ethyl acetate, fruits	MIC_2_	MIC_3_						
Water, stems	MIC_0_	MIC_2_	MIC_3_					
Water, leaves	MIC_3_							
